# Prevalence of sexual dysfunction in saudi women with Type 2 diabetes: Is it affected by age, glycemic control or obesity?

**DOI:** 10.12669/pjms.333.12166

**Published:** 2017

**Authors:** Turki A. AlMogbel, Hussein S. Amin, Saad M. AlSaad, Turky H. AlMigbal

**Affiliations:** 1 Turki A. AlMogbel, MBBS, ABFM. Buraydah Diabetes Center, King Fahad Specialist Hospital, Buraydah, Saudi Arabia; 2 Hussein S. Amin, MRCP(UK). Department of Family and Community Medicine, College of Medicine, King Saud University, Riyadh, Saudi Arabia; 3 Saad M. AlSaad, MBBS, SBFM. Department of Family and Community Medicine, College of Medicine, King Saud University, Riyadh, Saudi Arabia; 4 Turky H. AlMigbal, MBBS, SBFM. Department of Family and Community Medicine, College of Medicine, King Saud University, Riyadh, Saudi Arabia

**Keywords:** Diabetes mellitus, type 2, Diabetologist, Family medicine, Primary health care, Sexual dysfunction, Prevalence, Glycosylated hemoglobin, Saudi Arabia

## Abstract

**Objective:**

Sexual dysfunction (SD), as a diabetes mellitus (DM)-related complication, is common among patients having diabetes. This study aimed to ascertain the prevalence of SD in Saudi women with type 2 DM and to determine whether age, glycemic control, and obesity are associated with SD or not.

**Methods:**

A total of 275 Saudi women with type 2 diabetes took part in this cross-sectional study and filled out the Female Sexual Function Index through a fill-coded questionnaire in primary care clinics in King Khalid University Hospital, Riyadh, in the period between January 2013 and May 2013. The level of glycosylated hemoglobin and the body mass index were assessed to evaluate the DM control status and obesity among the patients.

**Results:**

SD was reported by 88.7% of the Saudi women with type 2 diabetes. The results showed a significant association between the presence of SD and the increase in age of patients at 92% in the age group above 50 years. Glycemic control did not show a significant association with SD. The obesity factor showed a slight increase in SD by weight, but it was not statistically significant.

**Conclusion:**

The prevalence of SD among the Saudi women having type 2 diabetes is high and increases with age. No association was found between SD and glycemic control.

## INTRODUCTION

About 387 million people in the world had diabetes mellitus (DM) in 2014 and by 2035, about 592 million people are expected to suffer from the disease, and the increase is expected to be 53% worldwide and 85% in the Middle East and North Africa.^1^ In the Middle East and North Africa, one in ten adults has DM, and Saudi Arabia is the country with the seventh highest prevalence of DM at 23.9%.^1^ Moreover, the major increase in DM prevalence is expected to take place in developing countries by 2025.^2^ DM may lead to many difficulties in several areas of life, e.g., disturbance in sexual function.

In the 1950s, sexual dysfunction (SD) among diabetic men caught the attention of researchers, but SD in women remained entirely neglected until Kolodny’s article in1971.^3^ The association of SD with diabetic men has been established by a few studies in Saudi Arabia.^4^-^6^ The latest study indicated that 83% of Saudi diabetic men had erectile dysfunction (ED).^6^ Similar studies showed a high prevalence of ED in men with DM at 86%^4^, 75%.^5^

On the other end of the scale, the association of SD with women has not yet been well investigated in our community. Female sexual dysfunction is a common problem affecting 30%-78% of women in general.^7^ It has been estimated that 20% to 80% of women with diabetes have SD.^8^

Women are very self-aware when talking about sex in a professional setting.^9^ Sexual problems in women with DM mostly involve sexual desire, sexual satisfaction, orgasmic disorder, arousal disorder, and lubrication.^10^,^11^

As sexual health is a vital issue and patients may sometimes be embarrassed to inform their physicians about their complaints, this study attempted to add to the literature by filling this gap. This study aimed to determine the prevalence of SD in Saudi women diagnosed with type 2 DM and whether age of patients, glycemic control, duration of DM, and obesity are associated with SD or not.

## METHODS

A cross-sectional survey was used in this study. A total of 275 Saudi women diagnosed with type 2 DM who were registered in primary care clinics in King Khalid University Hospital, Riyadh, Saudi Arabia, participated by filling out questionnaires.

Participation in this study was completely voluntary and was done anonymously. The participants were told that they could quit completing the questionnaire at any time. Informed consent was provided with the questionnaire, and returning the questionnaire was voluntary. The participants were not promised to be given a reward after completing the questionnaire.

The participants were asked to fill out a questionnaire in the period of January 2013 to the end of May 2013 after selected in convenience procedure. Questionnaires with coded envelopes were given to the participants to encourage them to express their feelings honestly and to maintain their privacy.

The inclusion criteria were as follows: Saudi patient, 25 years of age or older, married, diagnosed with type 2 DM, and followed up in our clinics for at least a year (to check previous blood work). The exclusion criteria were chronic renal failure and illiterate people (because the patients had to answer questions about a sensitive issue by themselves).

The patients provided the following demographic information: patients’ age, profession, monthly income of the family (High: more than SR15000 “>$4000”, Middle: SR7500–SR15000 “=$2000–$4000” and Low: less than SR7500 “<$2000”), and educational level (Primary (grade 6 or less), Intermediate (grade 9), Secondary (grade 12), or Higher education (bachelor or postgraduate)).

The questionnaire included items on the duration of DM, the coexistence of other medical conditions (hypertension, ischemic heart disease, dyslipidemia, and psychological disorder), and DM medication. Other conditions, such as regular exercise, diet, and smoking, were recorded. The medical records and the pharmacy system were used to verify coexisting medical conditions and medication. By dividing the weight in kilograms (kg) by the squared height in meters (m^2^), the body mass index (BMI) was calculated. Thus, the patients were grouped into the following categories: normal weight (BMI = 18.5–24.9), overweight (BMI = 25–29.9), obese class I (BMI 30–34.9), and obese class II and more (BMI ≥ 35).^12^ Control of DM was identified by the glycosylated hemoglobin level (HbA1c). The patients were grouped into three according to their HbA1c: ≤ 7%, 7%–8.50%, and > 8.50%. This categorization is based on the fact that when HbA1c > 8.5%, the physicine is adviced to exchange the medicine that the patient is taking with another that is greater and more rapid glucose-lowering effectiveness, or potentially earlier initiation of combination therapy.^13^,^14^

SD was measured using a standard questionnaire. The Female Sexual Function Index (FSFI) consists of 19 questions grouped into six domains that assess desire, arousal, lubrication, orgasm, satisfaction, and pain during sexual intercourse.^15^ The Arabic translated version of FSFI was used to assess sexual functions in women. The FSFI questionnaire was first translated to Arabic and then retranslated to English to validate the translation. A pilot study was conducted to ensure that the participants completely understood the questions. Permission to use the Arabic translation was gained after the author’s review. Some words in some of the items were changed because of cultural issues, e.g., we used husband instead of partner. The internal consistency of our questionnaire was excellent with cronbach’s alpha equal 0.94. An FSFI score ≤ 26.55 out of 36 in all domains of the questionnaire was the criterion for accepting the presence of SD.^16^

The number of subjects was estimated on the basis of the mean of SD prevalence among type 2 DM in various studies, degree of precision (0.8), and level of significance (0.05).

SPSS software v. 21 was used to analyze the data.^17^ The purpose of the test (X^2^) was to verify the association between the prevalence of SD and the different risk factors. Fisher exact test was used when the conditions of the X^2^ test were not met. The odds ratio for individual factors was obtained as a measure of the correlation with SD. To evaluate the independent effect of every factor after controlling for potential confounders, substantial factors were exposed to a multivariate logistic regression analysis. P value of <0.05 was regarded as statistically significant.

The questionnaire was approved by Institutional Review Board (IRB) of the College of Medicine, King Saud University (Letter No. 12/3569/IRB).

## RESULTS

In the study period, 275 Saudi women diagnosed with type 2 DM filled out the questionnaire. The relation of SD to the demographic characteristics of the study sample is presented in Table-I. The results showed a gradual increase in SD by year from 74% in the age group less than 40 years to 92% in age group above 50 (p<0.001) (Table-I). No significant association was found between SD and duration of DM in the relation of SD to others factors (p=0.230)

**Table-I:** Demographic characteristics in and its association to Sexual Dysfunction(SD).

*Demographic Variables*	*No*	*Sexual Dysfunction*				*P-value*

		*With SD*		*Without SD*		

		*No (244)*	*%*	*No (31)*	*%*	
***Age***						
<40	43	32	74.4	11	25.6	0.005
40-49	106	96	90.6	10	9.4	
≥ 50	126	116	92.1	10	7.9	
Occupation*						
Employee	57	50	87.7	7	12.3	0.825
Housewife	199	177	88.9	22	11.1	
Retired	12	10	83.3	2	16.7	
***Educational level*** *						
Primary or less	80	76	95.0	4	5.0	0.084
Intermediate	58	51	87.9	7	12.1	
Secondary	64	52	81.3	12	18.8	
Higher education	59	52	88.1	7	11.9	
***Family income*** *						
Low	87	80	92.0	7	8.0	0.359
Middle	117	104	88.9	13	11.1	
High	50	42	84.0	8	16.0	
BMI*						
18.5-24.9	15	13	86.7	2	13.3	0.917
25-29.9	64	56	87.5	8	12.5	
30-34.9	75	66	88.0	9	12.0	
≥35	95	86	90.5	9	9.5	
***Duration of Diabetes*** *						
≤ 5 years	94	86	91.5	8	8.5	0.230
>5-10	66	61	92.4	5	7.6	
>10-15	50	41	82.0	9	18.0	
>15	42	36	85.7	6	14.3	
***Smoking***						
Yes	8	8	100.0	0	0.0	1.000
No	246	220	89.4	26	10.6	
***Co-morbid diseases***						
Dyslipidemia	194	176	90.7	18	9.3	0.106
Hypertension	118	108	91.5	10	8.5	0.203
IHD	9	7	77.8	2	22.2	0.269
Psychological Dis.	19	18	94.7	1	5.3	0.706
***Regular exercise***						
No	189	166	87.8	23	12.2	0.627
Yes	74	67	90.5	7	9.5	

Both family income and SD were inversely associated but were not significant (p=0.359). No clear association was found in the occupation factor, but the education factor was different. The relation between SD and women with primary education or less was higher (95%) than that between SD and women with secondary education (81%), but it was not significant (p=0.084). In the obesity factor, 13.3% of the normal weight sample according to BMI did not have SD, and 9.5% of the obese class II & more sample also did not have SD, but it was not statistically significant (p=0.917).

The prevalence of SD among Saudi women having type 2 diabetes was 88.7% according to the FSFI score. In the association between SD and HBA1c, 92% of the patients with HBA1c ≤ 8.5 had SD, and 83% of the patients with HBA1c > 8.5 had SD, but it was not statistically significant (p=0.092).

Moreover, 9% of patients diagnosed with hypertension did not have SD compared with 13% of the patients with no hypertension (p=0.20). About 9% of patients diagnosed with dyslipidemia did not have SD compared with 16% of patients without dyslipidemia (p=0.106).

We verified the patients’ medications and their relation to SD. In patients who used insulin alone or oral antidiabetic drugs, 7.7% and 9.4% of the patients did not have SD, respectively. About 18% of patients who used insulin and oral together did not have SD (p=0.267). The distribution of medication and its relation to SD was not significant in all medications, but the percentage of SD increased in patients who used b-blocker (p=0.338), calcium channel blocker (p=0.238), and statins (p=0.221). (Table-II).

**Table-II:** Distribution of the study population according to medication and Sexual Dysfunction (SD).

*Variables*	*No*	*Sexual Dysfunction*				*P-value*

		*With SD*		*Without SD*		

		*No (244)*	*%*	*No (31)*	*%*	
***Diabetes Medication***						
Diet	35	32	91.4	3	8.6	0.267
Oral	160	145	90.6	15	9.4	
Insulin	13	12	92.3	1	7.7	
Insulin + Oral	67	55	82.1	12	17.9	
***Statin***						
Yes	178	161	90.4	17	9.6	0.221
No	97	83	85.6	14	14.4	
***Aspirin***						
Yes	103	89	86.4	14	13.6	0.347
No	172	155	90.1	17	9.9	
***B-blocker***						
Yes	28	27	96.4	1	3.6	0.338
No	247	217	87.9	30	12.1	
***CCB’s***						
Yes	18	18	100.0	0	0.0	0.238
No	257	226	87.9	31	12.1	
***Diuretic***						
Yes	36	33	91.7	3	8.3	0.778
No	239	211	88.3	28	11.7	
***ACEI or ARB***						
Yes	130	115	88.5	15	11.5	0.895
No	145	129	89.0	16	11.0	
***Antidepressant***						
Yes	20	18	90.0	2	10.0	1.000
No	229	204	89.1	25	10.9	

In the univariate analysis, an association was found in examining one factor at a time (Table-III). Moreover, as age increased, the odds ratio (OR) also increased. In consideration of the age group < 40 as reference for the age factor (OR of 1), the age group 40–49 had OR of 3.30 (p<0.01) and the age group >50 had OR of 3.99 (p<0.005). In BMI, OR also increased as the weight increased. In consideration of normal weight as the reference (OR of 1), overweight had OR of 1.08 (p=1), obese class I had OR of 1.13 (p=1), and obese class II and more had OR of 1.47 (p=0.644). The relation is different in glycemic control (HbA1c). OR in patients with HBA1c >7–8.50 was 1.10 (p=0.870) and that in patients with HbA1c >8.50 was 0.45 (p=0.074). Prevalence of SD was the lowest (83.6%) in the less glycemic control group (>8.5 of HbA1c), as compared to 91.8% and 92.5%, in ≤ 7 and >7-8.5 glycemic control groups respectively.

**Table-III:** Results of univariate analysis of factors associated with (SD).

*Variables*	*Odds ratio*	*P-value*
***Age***		
<40	1	Ref
40-49	3.30	0.010
≥50	3.99	0.002
***Education Level***		
Higher Education	1	Ref
Secondary	0.583	0.295
Intermediate	0.981	0.973
Primary or less	2.558	0.150
***Gylcemic control (HbA1c)***		
≤ 7	1	Ref
>7-8.5	1.10	0.870
>8.5	0.45	0.074
***BMI***		
18.5-24.9	1	Ref
25-29.9	1.08	1.000*
30-34.9	1.13	1.000*
≥35	1.47	0.644
Duration of Diabetes (in years)*		
≤ 5	1	Ref
>5-10	1.13	0.831
>10-15	0.42	0.093
>15	0.56	0.306

In the duration of DM, the duration < 5 years was considered as reference (OR of 1). Duration of 5–10 years had OR of 1.13 (p=0.831), duration of 10–15 years had OR of 0.42 (p=0.093), and duration of more than 15 years had OR of 0.56 (p<0.306). Co-morbid disease was not found to be significantly associated with sexual dysfunction, e.g., hypertension (OR:1.67, p=0.203), IHD (OR:0.43, p= 0.269), and dyslipidemia (OR:1.87, p=0.106).

Multivariate logistic regression analysis indicated that the odds ratio for the factors remained significant. The results regarding age were not significant (OR:0.525, p<0.108) because two age groups (<50 years and ≥ 50) were used. However, the duration of DM remained significant (OR:3.187, p <0.01) (Table-IV).

**Table-IV:** Results of multivariate analysis of factors associated with Sexual Dysfunction(SD).

Duration of Diabetes (in years)*	*No*	*Sexual Dysfunction*				*Exp (B)*	*P-value*

		*With SD*		*Without SD*			

		*No (244)*	*%*	*No (31)*	*%*		
≤ 10	160	147	91.9	13	8.1	3.187	0.010
>10	92	77	83.7	15	16.3		

## DISCUSSION

The prevalence of SD among the Saudi women with type 2 diabetes was 88.7%. Ziaei-Rad et al., indicated that 88% of Iranian women had SD.^18^ Mezones-Helguin et al., found that the prevalence of SD among women was 75%, and Ogbera et al., argued that 88% of women had SD.^19^,^20^ Esposito et al., demonstrated that 53.4% of diabetic women had SD.^21^ Abu Ali et al., reported that 59.6% of Jordanian women with types 1 and 2 DM had SD.^22^

The differences in prevalence rates in previous studies could be attributed to the following factors: studied population, methods used to assess SD, age groups, andsample size. Two more factors also lead to different prevalence rates: First, the different cut-off values in studies using the same scale (e.g., Esposito et al.^21^ used a score of 23 out of 36 as the cut-off for diagnosed SD in FSFI and we used 26.55 out of 36 in our study for the same scale)^16^. Second, the methods used to ensure privacy as the subjects were asked to discuss sensitive issues (e.g., coded envelopes for the participants in our study).

No association was found between glycemic control and the prevalence of SD in our study similar to the studies of Esposito et al.^21^, Abu Ali et al.^22^, and Elyasi et al.^23^ However, this finding is contrary to that of Ziaei-Rad et al.,^18^ which explored SD in both genders and both types of DM. Also, El-Sakka et al.^24^ show a significant association in its study that was on male diabetic patients. That association was between the number of patients with low level of total testerone and poor control DM, and that patients with low level of testerone were two times more likely to have severe ED. A significant associations between control of DM and normal level of total testerone at 3- and 6- month follow up visits was showed in other study.^25^

Age factor had a significant association with the prevalence of SD. The age groups factor showed a gradual increase in SD by year from 74% in the age group less than 40 years to 92% in the age group *above 50*. However, higher age groups experienced elevated rates of SD in Ziaei-Rad et al.’s study,^18^ but no significant statistical relationship was found between age and SD. In the duration of DM and its effect on SD, no significant association was found between SD and duration of DM in our study sample. Ziaei-Rad et al.^18^ and Esposito et al.^21^ did not report any major differences between duration of DM and SD in both genders. However, some studies (e.g., Abu Ali et al.^22^ and Mazzilli et al.^26^) found a significant association between SD and duration of DM.

In the obesity factor, a gradual increase in SD by weight was found, but it was not statistically significant similar to Elyasi et al.^23^ This finding was contrary to that of Esposito et al., who found an association between obesity and SD.^21^ Itr was also contrary to El-Sakka et al.^24^ study that showed a significant association between the increase in BMI and the presence of low testosterone level in group of patients, that was two times more likely to have severe ED than patients with normal testosterone level in same study.

The strengths of this study are the use of a validated measure of SD, a cut-off level of FSFI score accepted in the cross-validation study^16^, and a relatively large number of subjects investigated.

### Limitations of the study

First our study design is cross sectional which is not the best design to test the association between the SD among patients with diabetes and glycemic control. Second, our sample size was convenience sample and taken from a single institute which make the generalizability difficult. Lastly, The lack of non-diabetic control group to compare with was one of the limitations for this study that must overcome in future studies.

## CONCLUSION

SD prevalence was high in Saudi women having type 2 diabetes. Moreover, a correlation was found between SD prevalence and age, but no correlation was found between SD and glycemic control. A gradual increase in SD by weight was observed, but it was not statistically significant. To the best of our knowledge, we report the first study on the prevalence of SD among Saudi women patients with type 2 DM.
